# Continuous Analysis of Running Mechanics by Means of an Integrated INS/GPS Device

**DOI:** 10.3390/s19061480

**Published:** 2019-03-26

**Authors:** Pavel Davidson, Heikki Virekunnas, Dharmendra Sharma, Robert Piché, Neil Cronin

**Affiliations:** 1 Faculty of Information Technology and Communication Sciences, Tampere University, 33720 Tampere, Finland; heikki.virekunnas@tuni.fi (H.V.); dharmendra.sharma@tuni.fi (D.S.); robert.piche@tuni.fi (R.P.); 2 Neuromuscular Research Centre, Faculty of Sport and Health Sciences, University of Jyväskylä, 40014 Jyväskylä, Finland; neil.j.cronin@jyu.fi

**Keywords:** gait analysis, INS/GPS, machine learning, neural networks, sports equipment, velocity measurement

## Abstract

This paper describes a single body-mounted sensor that integrates accelerometers, gyroscopes, compasses, barometers, a GPS receiver, and a methodology to process the data for biomechanical studies. The sensor and its data processing system can accurately compute the speed, acceleration, angular velocity, and angular orientation at an output rate of 400 Hz and has the ability to collect large volumes of ecologically-valid data. The system also segments steps and computes metrics for each step. We analyzed the sensitivity of these metrics to changing the start time of the gait cycle. Along with traditional metrics, such as cadence, speed, step length, and vertical oscillation, this system estimates ground contact time and ground reaction forces using machine learning techniques. This equipment is less expensive and cumbersome than the currently used alternatives: Optical tracking systems, in-shoe pressure measurement systems, and force plates. Another advantage, compared to existing methods, is that natural movement is not impeded at the expense of measurement accuracy. The proposed technology could be applied to different sports and activities, including walking, running, motion disorder diagnosis, and geriatric studies. In this paper, we present the results of tests in which the system performed real-time estimation of some parameters of walking and running which are relevant to biomechanical research. Contact time and ground reaction forces computed by the neural network were found to be as accurate as those obtained by an in-shoe pressure measurement system.

## 1. Introduction

Contemporary systematic approaches for the analysis of walking and running mechanics use a combination of high-speed video analysis and ground reaction force and EMG measurements, which can provide relatively detailed gait information including the involved forces, pressure distribution, joint angles, running velocity, and other characteristics. However, the limitations of such approaches include high cost, lack of portability, lengthy analysis time, and the need for trained personnel.

A strong need exists for small and unobtrusive equipment that still provides meaningful information about athletic performance in the field without unnecessarily encumbering the athlete or constraining natural movement. It should also be possible to make such information rapidly available to coaches and athletes, presented in a form that is practically relevant and easy to interpret [1]. It is well-established that such equipment enables more frequent and regular collection of data, providing a continuous and comprehensive picture of individual-specific training adaptation, rather than just occasional snapshots. The ability to collect data in the field would also expand the horizons of movement analysis for clinical applications and physical rehabilitation treatments [2,3].

Application of inertial measurement sensors is a promising approach for movement analysis [4,5,6,7], which can provide an alternative to the traditional tools. Previous studies have used inertial measurement units (IMU) to capture human motion [4], compute stride (a gait cycle, two consecutive steps) parameters [5], assess running performance [6], and detect the type of foot strike (rearfoot or forefoot) [7]. Some approaches require foot-mounted IMUs [8] or multiple IMUs [9,10]. Inertial sensors can be used, for example, to detect temporal or spatial features of gait, such as the sharp peaks occurring when the foot hits the ground. Most publications consider only simple parameters, such as the stride time, that can be detected by accelerometers attached to the foot [8], thigh [11], and waist [12], or a combination of foot, shank, and thigh accelerometers [13]. Some studies have also used gyroscopes attached to the foot and shank to estimate the stride duration [14]. When sensors are placed on both legs, it is possible to analyze both step time and gait symmetry [15].

Some gait features, such as stride length, ground contact time (GCT), and ground reaction force (GRF) can be estimated indirectly based on the time series of the measured body and foot acceleration. These methods usually use regressions or artificial neural networks to compute the relationships between the acceleration vector and gait features [16,17,18,19]. However, due to the physiological differences between people, these methods require calibration or training data for each person to estimate gait features with reasonable accuracy [20].

Consumer devices for monitoring running performance include GPS tracking and accelerometer-based systems to count strides, step length, ground contact time, and vertical oscillation. These simple devices provide useful information about running duration, distance, and velocity, but lack accurate and detailed information about many key aspects of running techniques [21].

The IMU-based methods don’t provide all of the parameters that are required for running mechanics and kinematic analysis (e.g., these methods cannot compute speed). Their typical accuracy, in measured orientation (1–2°), is insufficient for kinematic analysis [22,23]. Additionally, in wireless IMUs, data transfer to the host computer is not reliable [9].

The aim of this paper is to present a measurement set-up based on an inertial navigation system (INS) combined with GPS (INS/GPS) to perform continuous 3D analysis of gait mechanics and technique during a session of outdoor running and walking on level terrain. This approach provides a range of important parameters that could have numerous applications in biomechanics research, such as detailed step characteristics, in-depth running mechanics, running efficiency, and inter-individual responses to fatigue. The current version works only for walking and running on level terrain. Other activities and types of terrains are excluded from the consideration. The data segments recorded during irregular motion (neither walking nor running) are discarded.

The main novelty of our approach lies in two key areas: (a) The comprehensive range of parameters that we compute gives more extensive information about running performance, compared to existing methods such as motion analysis and force platforms alone, and allows unimpeded movement; and (b) we compute these parameters with high precision and at high frame rates, making the approach suitable for research-grade experiments, as opposed to many commercial devices which only give a low-resolution overview of exercise performance.

## 2. Methods

This section describes the measurement equipment and data processing issues, including gait segmentation, computation of running/walking metrics, vertical velocity, and displacement computation, and the prediction of GCT and GRF using only body-mounted sensors.

### 2.1. Measurement Setup

**Figure 1:**
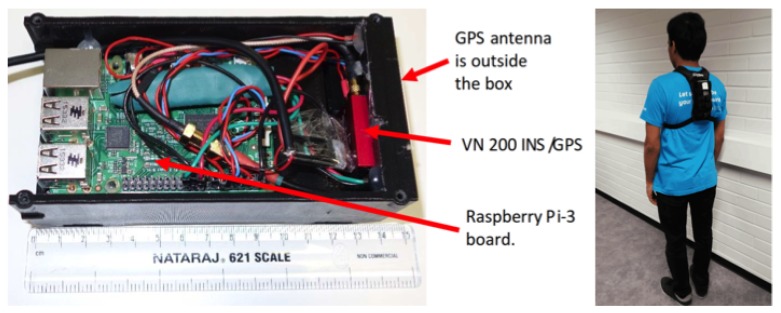
The data logger includes a Vectornav VN-200 inertial navigation system (INS)/GPS, a GPS antenna, a Raspberry Pi 3, and a battery. The battery is under the board. The data logger placement during field tests is on the right image. The GPS antenna is pointing upwards.

The measurement setup consists of a Raspberry Pi 3 model B board running a Linux OS, a Vectornav VN-200 GPS-aided inertial navigation system (INS/GPS), a GPS antenna, and a 4200 mAh power bank (see Figure 1). The VN-200 INS/GPS is connected to the board through an UART (universal asynchronous receiver/transmitter) serial connection. The GPS antenna is located in close proximity to the INS, making the lever arm error negligible. All components are packaged in a 3D-printed case. The data from the INS/GPS are stored on a memory card.

After the exercise, the data is transmitted to cloud storage using a 4G/LTE USB modem connected to the Raspberry Pi. The data analysis is done on a computer after the exercise. It is technically possible to send the data to the running coach during the exercise by 4G/LTE, but this is not implemented in the current version of our data logger.

This is a small (150 × 75 × 48 mm${}^{3}$, about 400 g), completely autonomous, and self-contained data logger that can be used in different field tests to measure many types of movement, including walking and running. The standard 4200 mAh power bank can provide power to the data logger for 4–5 h. The cost of components in our prototype is about 2500 euro. However, the cost (and size) can be reduced if, instead of the Raspberry Pi, a system on chip (SoC) is used and, instead of the rugged version of the VN-200, we use a surface mounted device or another less-expensive alternative. The size is mostly limited by the battery. If the power consumption is optimized, it can be reduced to about one watt, so an 1800 mAh battery will be enough to provide power for 5 h. In our outdoor walking tests, the unit was attached to the torso and the test route was on a level outdoor track.

The INS/GPS data are computed in the geographical coordinate frame with North, East, and Down axes. The output parameters are sampled at 400 Hz and include position, velocity, acceleration, orientation, angular velocity, and ground track (the path on the Earth’s surface). Each individual VN-200 INS/GPS sensor undergoes a robust calibration and acceptance testing process at VectorNav’s manufacturing facility. The accuracy specifications for real-time applications provided by the manufacturer are [24]:

Velocity accuracy: ±0.05 m/s
Heading, true inertial: 0.3° RMS
Pitch/Roll: 0.1° RMS
Angular resolution: <0.05°
Repeatability: <0.1°

This accuracy can be achieved when there is good GPS signal without multipath. Accurate heading estimation requires movement at a speed greater than approximately 1.5–2 m/s. At stand-still, the heading accuracy drops to approximately 1–2°, depending on the magnetic environment.

The computation of orientation in an integrated INS/GPS is completely different from that in an IMU. The fusion of INS and GPS is usually implemented using an error-state extended Kalman filter (EKF), whose states include at least errors of position, velocity, orientation, gyro drifts, and accelerometer biases (15 states). In INS, the orientation errors are observable through the velocity measurement orientation errors, and the gyro drifts can be estimated using GPS velocity measurements. The IMUs that are usually used in biomechanical research are, in fact, attitude and heading reference systems (AHRS). In AHRS, the vertical angles (pitch and roll) are usually corrected using the gravity vector, and heading is corrected using a magnetometer. In a typical high-end MEMS (microelectromechanical systems) IMU/AHRS (e.g., Xsens MTx), the orientation errors are about 1–2°. These errors can be larger when strong magnetic disturbances are present or during continuous high-dynamic applications. An integrated INS/GPS does not exhibit these problems. For the same accelerometers and gyros, the error in pitch and roll is about 0.1–0.2°, and 0.3° for heading. Magnetic disturbances don’t affect the accuracy. High dynamics have little impact on the performance. As is standard for human gait analysis, velocity and acceleration are computed in the anatomical frame, in which the *x*-axis is pointing in the anterior direction (direction of progression), the *z*-axis is vertical (parallel to the field of gravity) and points upwards, and the *y*-axis is perpendicular to the *x*- and *z*-axes and completes a right-hand triple.

**Figure 2:**
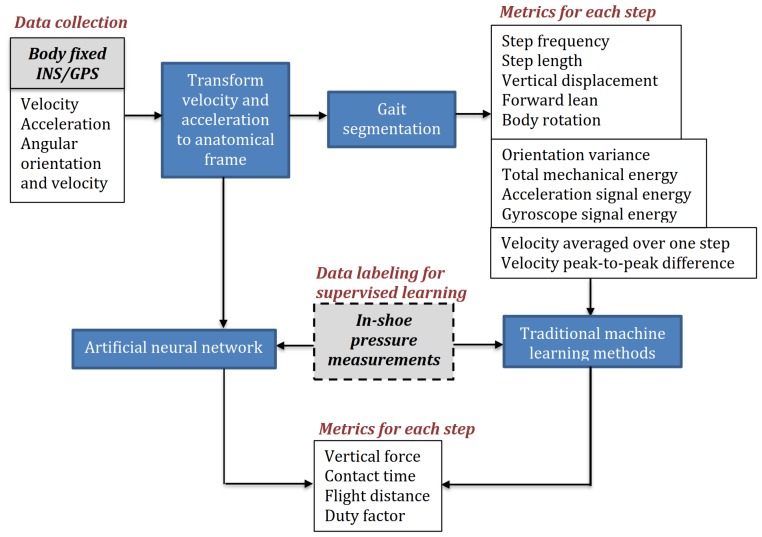
The system architecture. An optional in-shoe pressure measurement system provides a dataset for training and validation of machine learning methods that can be used for indirect estimation of ground contact time (GCT) and ground reaction forces (GRF).

Some parameters, such as ground contact time (GCT) and ground reaction forces (GRF), can be computed using machine learning methods and, therefore, require additional in-shoe pressure measurement equipment or multiple IMUs to create datasets for training and validation. The general system architecture, including instrumented insoles and/or force plates, is shown in Figure 2.

### 2.2. Data Processing

**Figure 3:**
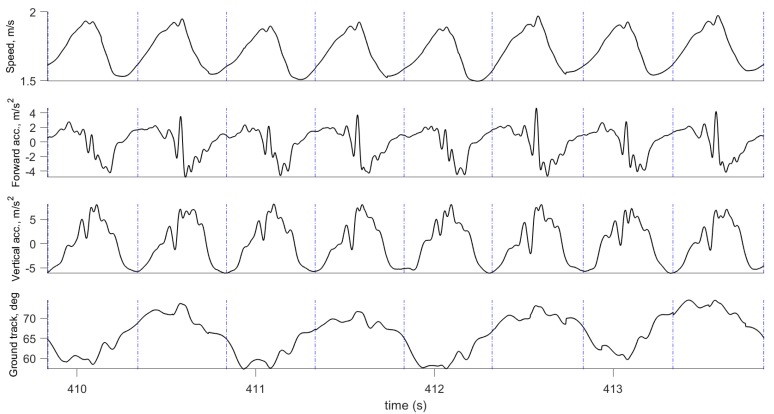
Motion parameters computed by the INS/GPS system for walking: Speed, forward and vertical accelerations, and ground track. Vertical lines show the beginning of the gait cycle.

**Figure 4:**
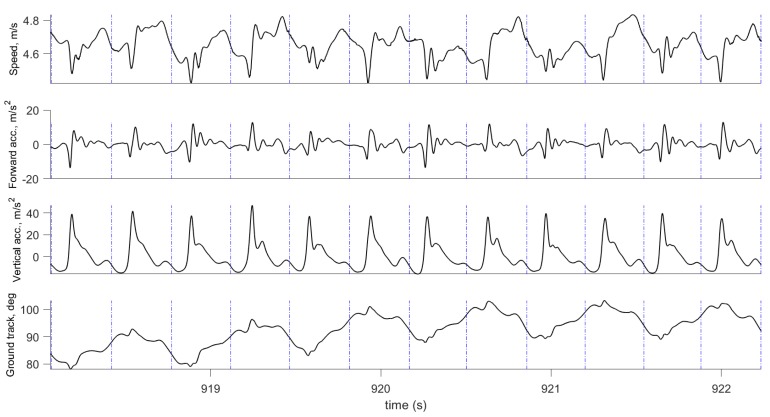
Motion parameters computed by the INS/GPS system for running: Speed, forward and vertical accelerations, and ground track. Vertical lines show the beginning of the gait cycle.

Additional data processing includes transformation of velocity and acceleration to the anatomical frame, step segmentation, computation of running metrics, and optional velocity and orientation accuracy improvement in post-processing. For running and walking applications, transformation of velocity and acceleration to the anatomical frame is accurately approximated by computing horizontal speed and the direction of movement, which is sometimes called the ground track. The following equations can be used to approximate speed and ground track:

(1) $$\begin{array}{cc} V(t) & =\sqrt{V_{N}{\left(t\right)}^{2}+V_{E}{\left(t\right)}^{2}} \end{array}$$

(2) $$\begin{array}{cc} \Psi (t) & =atan(V_{E}\left(t\right)/V_{N}\left(t\right)), \end{array}$$

where $V_{E}\left(t\right),V_{N}\left(t\right)$ are the East and North velocity components, V(t) is speed, and Ψ(t) is the ground track. In a similar way, acceleration is transformed to forward acceleration by computing the magnitude of the horizontal acceleration components. Examples of computed speed, ground track, and forward and vertical acceleration are shown in Figure 3 and Figure 4.

**Figure 5:**
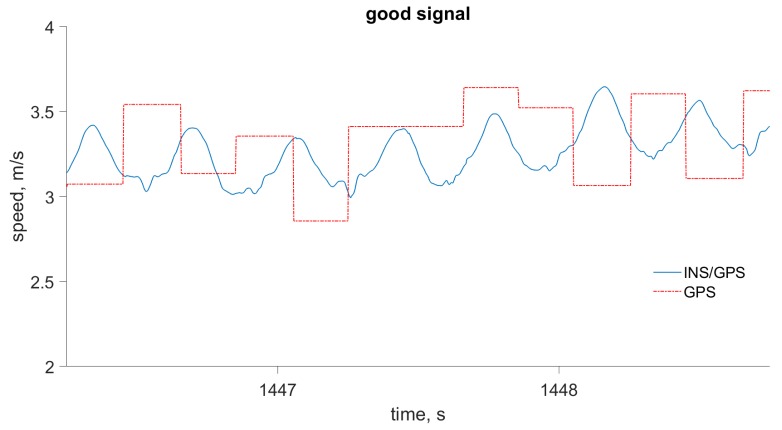
Walking speed computed by the INS/GPS integrated system and by the GPS receiver only. The plot shows the typical velocity accuracy for a consumer-grade single frequency GPS receiver.

**Figure 6:**
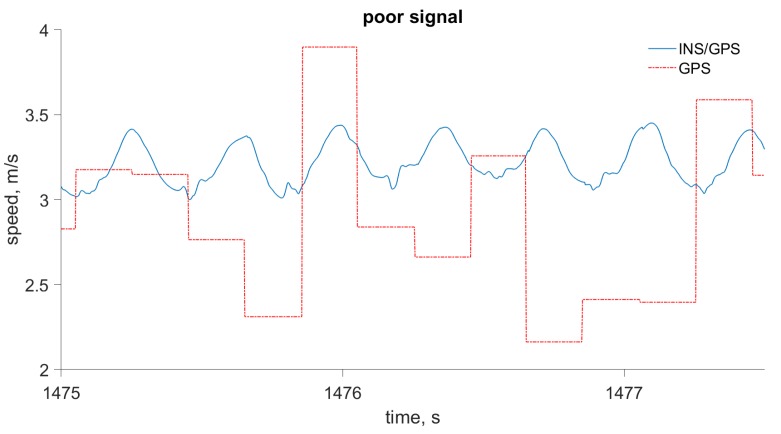
Walking speed computed by the INS/GPS integrated system and by the GPS receiver only. The plot shows degraded velocity accuracy for the same GPS receiver.

The advantages of INS/GPS for velocity computation, compared to a typical single-frequency GPS receiver with 5 Hz output rate, are demonstrated in Figure 5 and Figure 6. In Figure 5, the GPS velocity error is typical of the kind of consumer-grade receivers used in wearable devices for sport and well-being monitoring, when signal conditions are optimal. In Figure 6, the GPS velocity error is greater; this may be caused by multipath errors or the obstruction of some of the GPS satellites. In both cases, the combined INS/GPS solution has better accuracy and time-resolution: It can accurately follow the actual movement at an output rate of 400 Hz.

The combined solution accurately computes motion features that may be useful in gait analysis. These features are valuable when machine learning (especially deep learning) methods are used to indirectly estimate some gait parameters, such as initial contact and toe-off. The accuracy of combined INS/GPS can be improved in post-processing, for example, by applying the Bayesian smoothing procedure described in [25].

### 2.3. Gait Segmentation

There are many different approaches to human gait segmentation [26,27]. These approaches usually use body- or foot-mounted accelerometer measurements [12,28] to identify gait phases, such as touchdown or toe-off. The timing accuracy of these approaches is about 10–15 ms [29]. However, their reliability and robustness with respect to changing walking and running conditions or different gait styles is not very good.

**Figure 7:**
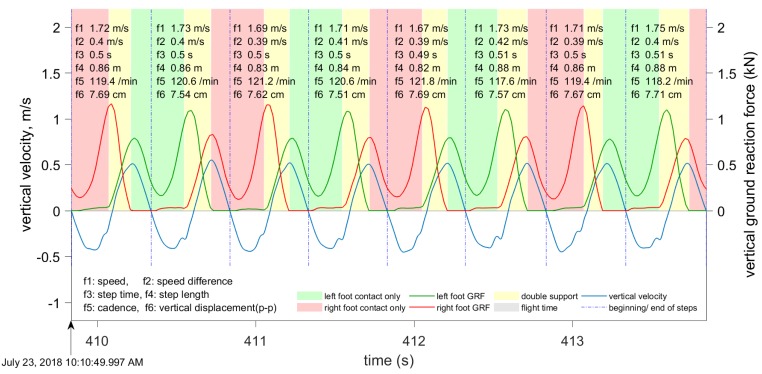
The segmentation of gait during walking, based on the vertical velocity. The plot shows the vertical velocity (blue), left (green), and right (red) foot force measurements. The following six metrics are displayed for each step: Speed averaged over one step, speed difference peak-to-peak, cadence, step length, step duration, and vertical displacement peak-to-peak.

**Figure 8:**
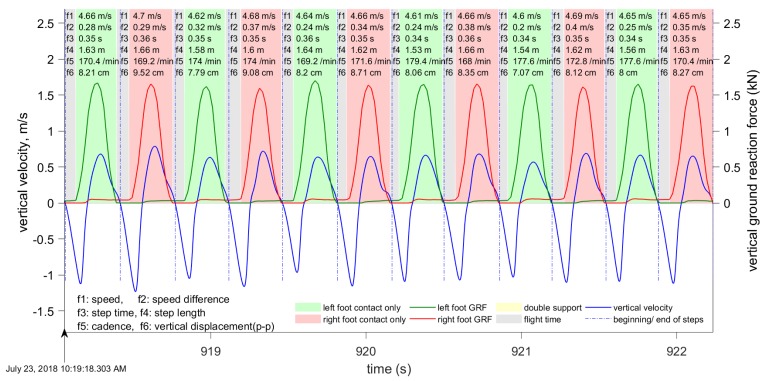
The segmentation of gait during running, based on the vertical velocity. Curves and metrics as in Figure 7.

Our approach towards gait segmentation is based on the periodicity of the vertical velocity. We define the beginning of each step to be the instant when vertical velocity changes from positive (center of mass (CoM) moving upwards) to negative (i.e., the instant when the CoM is at its highest). When vertical velocity falls to zero again and the CoM position is highest, this instant is close toe-off, and this is especially accurate in running. Figure 7 and Figure 8 show the results of gait segmentation.

The algorithm for gait segmentation requires pre-processing to remove the low-frequency drift in the vertical velocity caused mainly by accelerometer bias or due to walking/running on an inclined surface. We used a high-pass filter to remove the low-frequency velocity drift. This was implemented using the MATLAB Signal Processing Toolbox highpass function, which uses a minimum-order filter with defined stopband attenuation to compensate for the delay introduced by the filter. The following function parameters were chosen: Normalized passband frequency (wpass), 0.005π rad/sample; attenuation (stopbandattenuation), 30 dB; and steepness (steepness), 0.7.

**Figure 9:**
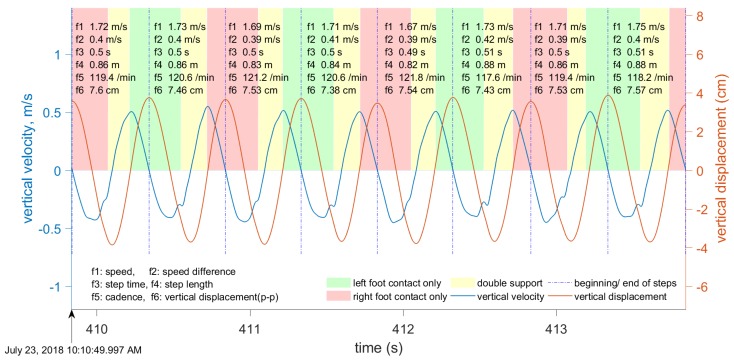
Vertical velocity (blue) and displacement (red) during walking. The following six metrics are displayed for each step: Speed averaged over one step, speed difference peak-to-peak, cadence, step length, step duration, and vertical displacement peak-to-peak.

**Figure 10:**
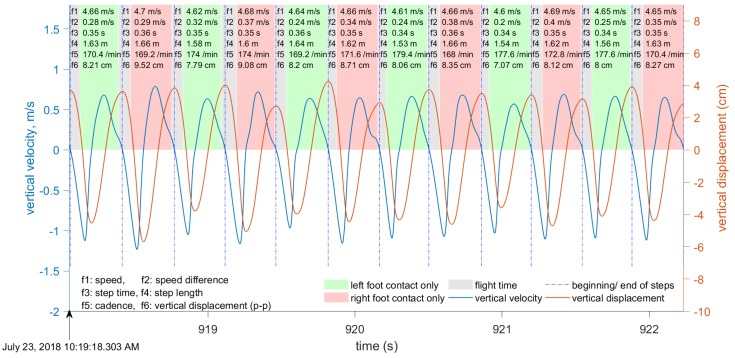
Vertical velocity (blue) and displacement (red) during running. The displayed metrics are as in Figure 9.

The input to the gait segmentation algorithm is a data window of the filtered vertical velocity data. In our experiments, the length of this window varied significantly, from 4 to 450 s, and the step segmentation accuracy was not affected. However, this data window must not include any irregular motion (i.e., motion that is neither walking nor running). The first stage is to determine the points where the vertical velocity is close to zero; that is, below the pre-defined threshold. During one step, the vertical velocity is equal to zero twice: Once when the CoM is closest to the ground, and again when it is furthest from the ground. The local maxima and minima in the CoM vertical displacement are at these points where the vertical velocity is zero (Figure 9 and Figure 10).

### 2.4. Computation of Running/Walking Metrics for Each Step

Features or metrics are computed for each step to facilitate analysis of the data. Our system computes the following metrics, which are commonly used in walking and running:

**Step frequency or cadence**: The reciprocal of step duration.
**Step length**: The distance travelled during one step.
**Forward lean**: The angle between the vertical axis and the body axis in the sagittal plane.
**Body rotation**: The amplitude of rotation around the vertical axis.
**Vertical displacement**: The peak-to-peak difference in the vertical movement.
**Speed averaged over one step**: Arithmetic mean of the speed (=step length divided by duration of step).
**Speed averaged over 10 m, 20 m, 50 m, and so on**.
**Contact time**: Stance phase duration.
**Duty factor**: The percentage of the time that the foot is on the ground.
**Flight distance** (only in running): The distance covered when neither foot is touching the ground.
**Vertical ground reaction force**.

The following metrics are also computed:

**Total mechanical energy**: The sum of kinetic energy and potential energy.
**Peak-to-peak speed difference during one step**.

Some metrics are computed directly from the available data in the INS/GPS output. However, vertical displacement, contact time, flight time, duty factor, and ground reaction forces require additional signal processing procedures. The computational procedures for these parameters are described in later sections. The computation of running and walking metrics requires that the data be segmented into individual steps. Our approach for gait segmentation, described earlier, is slightly different from the commonly-used approach of starting a gait cycle from initial contact. However, as explained in the next section, we found that the computed metrics are almost invariant to small shifts in the beginning of the gait cycle. Although all of the above-mentioned metrics are computed, only six metrics are shown in Figure 7, Figure 8, Figure 9 and Figure 10: Speed averaged over the step, peak-to-peak speed difference, step duration, step length, cadence, and vertical displacement.

### 2.5. Robustness of the Computed Metrics to the Beginning of the Gait Cycle

As it is not always possible to determine the touchdown and toe-off instances without an in-shoe pressure measurement system, we performed experiments to examine the effect of initiating the gait cycle at different instances on the computed metrics. The baseline was computed using the step segmentation approach described in the previous section, whereby the gait cycle was initiated at the moment of zero vertical velocity. The beginning and end of each step were then shifted by some value (e.g., 5% of the average step duration) and the metrics were recomputed for each step. The difference in the value of each metric was computed for each step, and mean and standard deviation were then computed. The process was repeated for shifts from ±5% to ±30% in steps of 5%.

**Figure 11:**
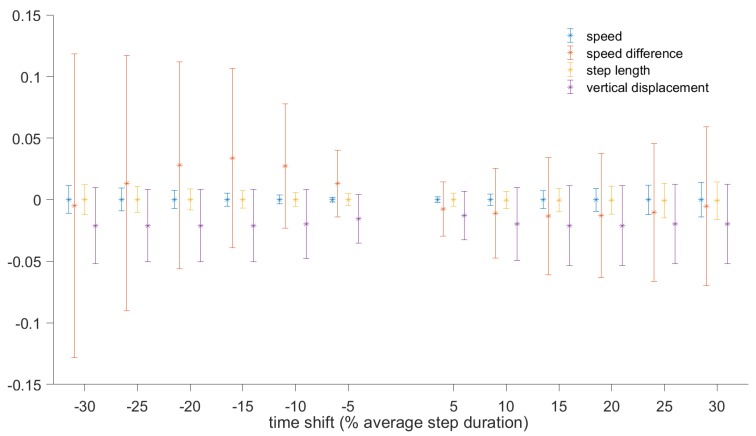
Relative error in speed, speed difference, step length, and vertical displacement to the beginning of the gait cycle. The results are based on 500 steps during walking.

**Figure 12:**
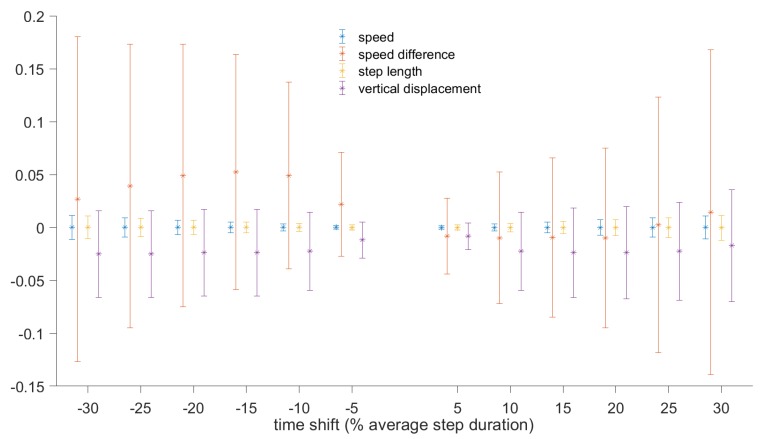
Relative error in speed, speed difference, step length, and vertical displacement caused by a shift in the gait cycle beginning. The results are based on 1000 steps during mix of walking and running.

Figure 11 shows the changes in the metrics (mean and standard deviation) for walking, based on 500 steps. Figure 12 shows the changes for running and walking, based on 1000 steps. It can be seen that the speed averaged over one step and step length are very robust to the instance when the gait cycle starts, with the errors below 2% of the baseline values. In terms of absolute values, the changes in step length do not exceed 12 mm. Changes in vertical oscillation do not exceed 5% (or 6 mm). Speed difference is the metric that is most affected by the shift in the beginning of the gait cycle, especially if the shift exceeds 10% of the gait cycle. For shifts exceeding 10%, the error in speed difference is about 10%, but in absolute values it does not exceed 0.05 m/s.

### 2.6. Vertical Displacement Estimation

Vertical displacement is measured because it gives an indication of how much work is required to lift the centre of mass [30]. As work against gravity is energetically costly, vertical displacement can be used to better understand the running energetics. The vertical displacement was computed for each step by numerical integration of vertical velocity. We used the MATLAB function cumtrapz (cumulative trapezoidal numerical integration), which computes the cumulative sum of the vertical velocity multiplied by the sampling time.

Oscillations of the vertical movement were calculated around a nominal height (mid-point of oscillations), which was determined by calculating the average for the entire window (including an integer number of steps). Therefore, at the beginning of the gait cycle, the vertical displacement of the CoM was at the maximum (highest point), and it was at the minimum (lowest point) in the middle of the gait cycle (when vertical velocity was zero and the sensor was closest to the ground).

The accuracy of the computed vertical displacement can be assessed analytically. We performed tests where a person was walking on a level outdoor track. In this case, the average vertical displacement over one step was zero. Therefore, when the vertical displacement is computed, any non-zero output indicates an error that is mainly caused by accelerometer measurement errors. According to the manufacturer’s specification, the accelerometer error is characterized by in-run bias stability (<0.04 mg), linearity (<0.5% full scale), and noise density (0.14 mg/Hz). As the duration of one step is very short (approximately 0.5 s), the effect of noise (or random walk) can be neglected. The bias error is also small and its contribution to the vertical displacement error is around 1 mm. Most of the error is caused by non-linearity and, in the worst-case scenario, it does not exceed 5 mm and is normally smaller.

We computed the vertical displacement error for the data presented in Figure 9 and Figure 10. For the eight steps shown in Figure 9, the vertical errors are (0.32, −0.29, 0.46, −0.56, 0.68, −0.39, 0.6, −0.32) mm. For the 12 steps shown in Figure 10, the vertical errors are (−0.89, 2.25, −3.22, 4.11, −3.78, 1.86, −0.0, −0.70, 0.43, 0.12, −0.87, 1.93) mm. As expected, the errors are larger for running, with a maximum recorded error of 4.11 mm. The vertical errors for walking are below 1 mm and, therefore, can be neglected. The same time interval of four seconds was used for both walking and running. Therefore, the amount of steps is different because the cadence during walking is different from the cadence during running.

### 2.7. Indirect Estimation of Ground Contact Time and Ground Reaction Forces

The data logger can be used for estimation of some parameters, such as GCT and GRFs, which are not measured directly. At present, these parameters can be measured only by specialized equipment, such as an in-shoe pressure measurement system or force plates [31,32,33]. Using our data logger, these parameters can be estimated indirectly using machine learning. Measurements of GCT and GRF from the Moticon instrumented insoles are only used to create a training dataset and train the neural network. The training data contains simultaneous data records from the sensors (3D acceleration and 3D angular rates) and instrumented insoles. In this case, the insoles provide known GCT and GRF data which can be used to train the neural network. The training data have to be accurate and should include measurements with different speeds, shoes, and surface conditions. We trained our neural network for walking and running speeds from 1 m/s to 8 m/s on an all-weather running track and asphalt with three subjects and the same shoes.

**Figure 13:**
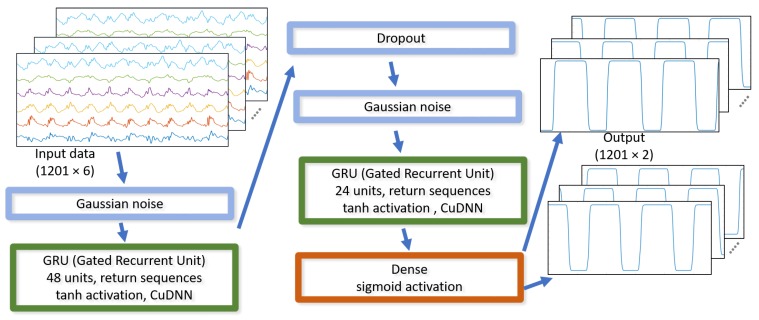
The recurrent neural network implementation.

We used a deep learning network regression algorithm, which was implemented in Google’s TensorFlow. For feature selection, we compared the classification accuracy with different feature sets, using the “leave one out” procedure, and concluded that out of 12 features (3d acceleration, 3d angular velocity, orientation quaternion, ground speed, and vertical velocity) only the six features (3d acceleration and 3d angular velocity) are important for classification accuracy. Based on experiments, we also found that, of these six features, the vertical and forward acceleration are the most important for the accuracy of the trained neural network. The addition of the other four parameters (lateral acceleration and angular velocity) only slightly improves the accuracy of GCT and GRF estimation. A block diagram of the algorithm is shown in Figure 13.

The basic network configuration consists of one or more recurrent layers of long short-term memory (LSTM) units and gated recurrent units (GRU), some noise/dropout layers to improve generalization, and a fully-connected dense layer with two independent outputs and sigmoid activation. For the GCT prediction, the input data (including all components of measured acceleration and angular velocity, sampled at 400 Hz) was divided into sequences of 1201 samples which overlapped by 50%. The input data to the neural network was scaled to range roughly from −1 to 1. The loss function was modified to ignore the beginning of the sequence, allowing the network to “see” some data before making meaningful predictions. Only the second half of the predictions in the sequence were used as the output. For the GRF prediction, the neural network structure is exactly the same as for GCT, except for the very last output layer, which has rectified linear unit (ReLU) as the activation function, instead of sigmoid.

The training time on a Nvidia GTX 1080 GPU is usually about 2–10 min, and it is the same for GCT and GRF. The computing time to make a prediction is around 10–20 s on the same GPU. After the training, the algorithm can predict both the GCT and GRF without using direct measurements of foot pressure. The evaluation data were not used for training, but were data from tests with the same subject.

**Figure 14:**
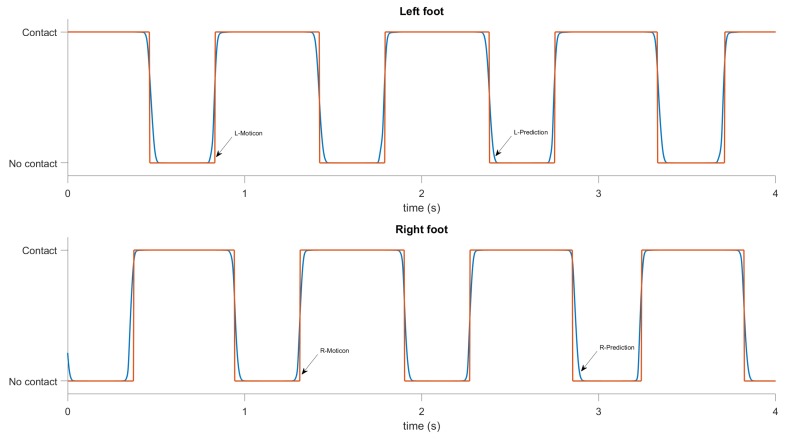
GCT predictions, computed by the recurrent neural network during walking. The upper plot shows the predicted (blue) and reference (red) ground contact for the left foot, and the lower plot shows the same for the right foot.

**Figure 15:**
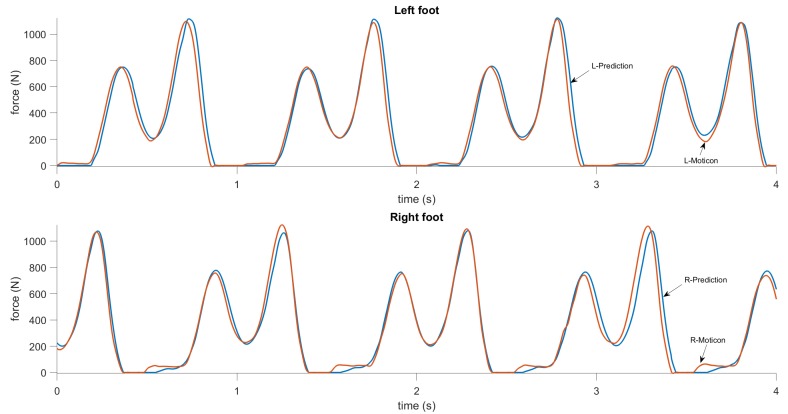
GRF predictions, computed by the recurrent neural network during walking. The upper plots show the predicted (blue) and reference (red) GRF for the left foot, and the lower plots show the same for the right foot.

The results for GCT and GRF prediction are shown in Figure 14 and Figure 15. The reference data from the Moticon insoles were post-processed to have a binary output for the ground contact (i.e., 0 if a foot is not touching the ground, 1 if a foot is on the ground). Figure 14 shows the results for the ground contact measurements and predictions. It can be seen that the predicted curve accurately approximates the measurements. Most of errors are during touchdown and toe-off instances, when the ground forces are small. The RMSE in GCT prediction is about 13 ms. The normalized RMSE for left and right foot are 2.95% and 2.88%, respectively. Figure 15 shows results for the vertical ground reaction force measurements and predictions. It can be seen that the predicted curve accurately approximates the measurements. The normalized RMSE in GRF prediction for mixed data (walking and running) for left and right foot are 4.48% and 4.83%, respectively.

## 3. Test Results

In this section, we describe the results of some running and walking tests, as well as performance comparisons with Moticon insoles and the Garmin HRM-Run chest strap, which computes some running metrics including vertical oscillation and GCT. During these tests, the subjects wore the data logger at the same time as Moticon insoles and, in some tests, the Garmin HRM-Run chest strap. The data from these three devices were synchronized.

### 3.1. Data Acquisition

We collected approximately six hours of data with the three subjects (more than 40,000 steps, approximately half of them while running) during several field tests on an outdoor track and around a university campus. The tests were done at a range of walking and running speeds (1–8 m/s) with three healthy young male adults. During these tests, the data logger was placed in a small backpack that kept the device in a stable position on the back (Figure 1). The data logger’s output (velocity, acceleration, quaternion, angular velocity, and so on) was synchronized using very accurate GPS time (the timing error was about $10^{-6}$ s). The Moticon insoles replaced standard insoles in Asics DS trainer 16 neutral running shoes, and provided the reference touchdown, toe-off, and vertical GRF. The data logger output data were synchronized with the Moticon insoles output. The Moticon output rate depended on the amount of pressure sensors that could be used. For the maximum amount of 13 sensors on each insole, the output rate was 50 Hz. If only 6 sensors on each insole were used, the output rate was 100 Hz. The Moticon’s data was interpolated and then sampled at a higher frequency (400 Hz) that matched the output rate of the other proxy variables. The Moticon foot force measurements are shown in Figure 7 and Figure 8.

In some tests, we also recorded data from the Garmin HRM-Run strap, which was synchronized using GPS time. The Garmin’s output rate was only 1 Hz. If there were several steps during the 1 s period the GCT, vertical oscillations, speed, and so on, were the same for these steps. The data was time stamped, based on GPS time. The Moticon’s data was synchronized with the data logger data using jumps and adjustments in post-processing. Fatigue and speed were not controlled.

In the introduction, we mentioned that one problem with wireless IMUs (e.g., Opal APDM, Xsens Mtw, and so on) is data losses during the wireless data transmission to the host computer. In our case, there was no wireless data transmission. The VN-200 INS/GPS was connected to the host computer (Raspberry Pi) directly, using a wire. As the data was time-stamped using GPS time, we always know whether some data was missing, but this never happened during our tests, because the system was very reliable.

### 3.2. Results

Figure 3 and Figure 4 show examples of the most important parameters recorded during these tests: Speed, vertical acceleration, magnitude of forward acceleration, and ground track. In addition to these parameters, the data logger measured upper body angular velocity and orientation. These data were segmented into steps using the procedure described in Section 2.3, and the beginning of each step cycle is indicated by a vertical line. The gait segmentation is required to compute the walking and running metrics for every step. Examples of metrics are shown in Figure 7 and Figure 9 for the same time interval as in Figure 3, and in Figure 6 and Figure 12 for the same time interval as in Figure 4. In these plots, the beginning of each step is at the point where the vertical velocity is zero and the device is closest to the ground. When the Moticon insoles are used, gait segmentation can begin from touchdown. However, touchdown and toe-off can be identified from the acceleration and ground track data using the machine learning technique described in Section 2.7, and the results of which are shown in Figure 14. In this case, an in-shoe pressure measurement system was used only during the training phase.

The speed and forward acceleration plots (Figure 3 and Figure 4) show subtle details of each step and the differences between steps. Significant changes in speed and forward acceleration within one step indicate significant braking forces and potentially incorrect technique. Ground track indicates body sway and step width. These are additional sources of important information about human gait that are often overlooked in many studies. Another important outcome is the gait segmentation and computation of metrics which significantly reduce the amount of data, and present real-time information in a readily accessible format for the coach and athlete. We also demonstrated that the timing accuracy of our gait segmentation approach can match the accuracy of instrumented insoles (Figure 14 and Figure 15).

### 3.3. Comparison with Garmin HRM-Run

We carried out field tests to compare GCT and vertical oscillation computed by the data logger to the corresponding values from the Garmin HRM-Run chest strap. Moticon insoles provided the reference data for GCT. Data were synchronized and recorded simultaneously on these three devices. A Garmin fenix 3 HR wristwatch recorded the data from the Garmin chest strap with a 1 Hz sampling rate. GCT and GCT balance were computed accurately only during running. The field tests included walking, jogging, and running on a 400 m outdoor track with synchronization jumps before and after each segment to correct the clock drift in the insole data.

**Figure 16:**
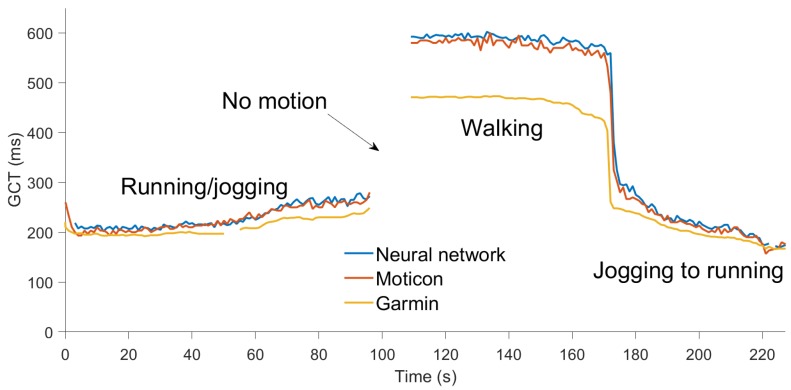
Ground contact time computed by Moticon insoles (red), the Garmin HRM-Run (amber), and indirect estimation using the neural network (blue) during walking, jogging, and running.

**Figure 17:**
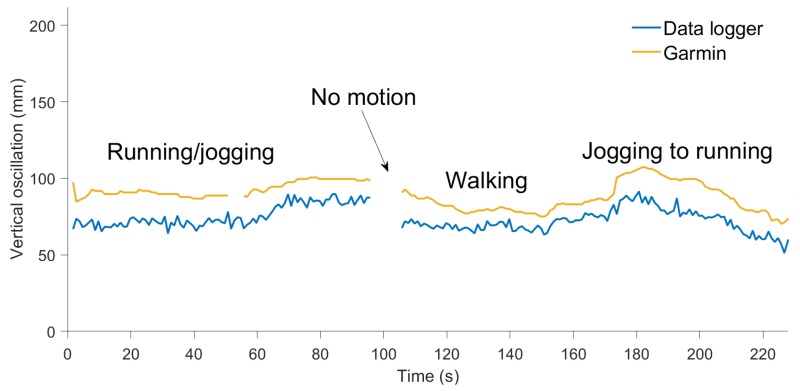
Vertical oscillation computed by the Garmin HRM-Run (amber) and by our device (blue) during walking, jogging, and running.

The Moticon GCT was calculated from the instant of heel touchdown to the instant of toe-off using measured force, which was sampled at 100 Hz. To match the Garmin’s output it was downsampled to 1 Hz. The GCT was also computed by the data logger using the neural network presented in Section 2.7, which was trained using previously collected Moticon data. Figure 16 and Figure 17 show the data from this field test. The absolute difference between GCT computed by Moticon and the data logger was around 12 ms, on average. The Garmin underestimated the GCT by about 25–30 ms for jogging and running, and by about 100 ms for walking.

Vertical oscillation was estimated from the data logger, using velocity based step segmentation, and was resampled to one second intervals to match the output from the Garmin. The results show that vertical oscillation computed by the Garmin HRM-Run during walking and running was 15–20 mm larger than the value computed by the data logger (Figure 17).

## 4. Conclusions

This paper describes equipment and a methodological approach that can be applied to biomechanical research and performance evaluation in sports. Our approach uses a new generation of small sensors to measure and analyze human movement in a natural environment with the ability to collect large volumes of ecologically-valid data. The INS/GPS data logger enables unconstrained monitoring of human gait in the field, and accurately computes several motion parameters, such as speed, forward and vertical accelerations, body orientation, and ground track. Its accuracy is comparable with that of gold standards such as optical motion tracking systems and force plates. The output parameters provide valuable information about the running and walking mechanics, and the device presented here could be used for various applications, including the study of gait variability, running technique, and gait asymmetry, among others.

The measurement accuracy of the velocity and angular orientation is better than in other IMU-based systems currently used in biomechanical research [6,34,35,36]. To present the data in a convenient format, gait segmentation is performed and a set of metrics is computed for each step. The following accuracy has been achieved:

- Errors in step length and vertical displacement (on flat surfaces) are less than 1 cm.
- Timing errors in gait segmentation and step duration are less than 5 ms.
- Speed error is about 0.05 m/s. Variations of speed within a step can be determined with an error that is smaller than 0.01 m/s.
- The normalized RMSE in GCT prediction, using only a body-mounted sensor unit, is less than 3%.
- The normalized RMSE in GRF prediction, using only a body-mounted sensor unit, is less than 5%.

It was also confirmed in the experiments that some metrics (speed averaged over the step, peak-to-peak speed difference, step duration, step length, cadence, and vertical displacement) are insensitive to the instant when the gait cycle starts. Thus, the gait cycle does not have to start at touchdown. This can simplify the gait segmentation procedure. To demonstrate that this measurement methodology can be used to generate useful features for indirect estimation of other important parameters, we have shown how the data can be used to train a recurrent deep neural network to predict the GCT and GRF using only a body-mounted sensor unit.

An optional human-computer graphical interface is shown in Figure 7, Figure 8, Figure 9 and Figure 10. The plots show only six metrics for each step, but other metrics can be also displayed. This information helps to analyze running technique systematically and can be used together with video analysis. The system is indispensable when the athlete is training without the coach and a form of self-assessment or reportable measurements is required.

The data measured by the data logger can be used as input to machine learning algorithms to compute parameters that are not measured directly, such as GCT, GRF, and touchdown and toe-off instances. The device computes GCT and vertical oscillation more accurately than the Garmin HRM-Run. Currently, the accuracy in GCT prediction using only a body-mounted INS/GPS system without instrumented insoles is about 12 ms, but we believe that this accuracy can be improved with further tuning of the algorithms. Potentially, machine learning could also be used to detect changes in technique because of injuries, fatigue, and motion disorders.

Our plans for future work include extensive testing of our device with different people and in different conditions, publishing an open database that can serve as a benchmark for different approaches, improvement of the machine learning method for indirect GCT and GRF prediction, and implementation of this approach in different applications (rowing, paddling, cross-country skiing, and so on). We believe that further development of this kind of new sensor technology, combined with machine learning, can lead to improvements in athletic performance, injury rehabilitation, and motion disorder diagnostics.

## Abbreviations

The following abbreviations are used in this manuscript:

AHRS	Attitude and heading reference system
CoM	Center of mass
CuDNN	The NVIDIA CUDA deep neural network library
EKF	Extended Kalman filter
GCT	Ground contact time
GRF	Ground reaction force
GPS	Global positioning system
GPU	Graphics processing unit
GRU	Gated recurrent units
HRM	Heart rate monitor
IMU	Inertial measurement unit
INS	Inertial navigation system
LSTM	Long short-term memory
LTE	Long term evolution
MEMS	Microelectromechanical systems
ReLU	Rectified linear unit
RMS	Root mean square
RMSE	Root mean square error
SoC	System on chip
UART	Universal asynchronous receiver/transmitter
